# Nontuberculous Mycobacteriosis as a Cause of Cervical Lymphadenopathy: A Retrospective Case Series

**DOI:** 10.3390/microorganisms14030545

**Published:** 2026-02-27

**Authors:** Anna Stenzl, Annette Runge, Anna Landegger, Felix Johnson, Florian K. Enzmann, Benedikt G. Hofauer, Teresa B. Steinbichler

**Affiliations:** 1 Department of Otorhinolaryngology, Medical University of Innsbruck, 6020 Innsbruck, Austria; anna.stenzl@i-med.ac.at (A.S.); annette.runge@tirol-kliniken.at (A.R.); felix.johnson@i-med.ac.at (F.J.); benedikt-gabriel.hofauer@i-med.ac.at (B.G.H.); teresa.steinbichler@i-med.ac.at (T.B.S.); 2 Department of Vascular Surgery, Medical University of Innsbruck, 6020 Innsbruck, Austria; florian.enzmann@i-med.ac.at

**Keywords:** nontuberculous mycobacteria, cervical lymphadenopathy, pediatric infection, ultrasonography, *Mycobacterium avium*

## Abstract

Background: The global incidence of infections caused by nontuberculous mycobacteria (NTM), environmental pathogens commonly found in soil and water, is increasing. Among the various NTM species, *Mycobacterium avium* is most frequently implicated. Clinical manifestations are diverse and include chronic pulmonary disease, disseminated infection, and cervical lymphadenopathy, particularly in children. This study aimed to evaluate the diagnosis and treatment of NTM-associated cervical lymphadenopathy at a tertiary referral center. Methods: All patients treated for NTM-associated cervical lymphadenopathy at the Department of Otorhinolaryngology, Medical University of Innsbruck, between 2010 and 2024 were retrospectively analyzed. Demographic data, imaging findings, microbiological results, therapy, and follow-up were evaluated. Results: A total of 22 patients with a median age of 1 (IQR 2) year were identified, most of whom originated from rural areas (91%). Diagnosis was based on characteristic clinical and imaging findings. Cervical ultrasonography was performed in all patients, and additional magnetic resonance imaging was performed in 55%. Microbiological confirmation was achieved in 64% of cases using liquid or solid cultures, while no pathogen was detected in 36%. Treatment consisted of combined antibiotic therapy with clarithromycin and rifampicin for 3–6 months, with additional surgical intervention in selected cases. Follow-up demonstrated clear regression of lymphadenopathy in the majority of patients within six months. Conclusions: NTM infection should be considered in the differential diagnosis of cervical lymphadenopathy. Pathogen detection remains challenging, and antimicrobial therapy alone may be sufficient in selected cases.

## 1. Introduction

The global incidence and prevalence of nontuberculous mycobacterial (NTM) infections are increasing, posing significant challenges to contemporary clinical practice and public health. These epidemiological trends are increasingly modulated by environmental factors, including climate change. NTMs are ubiquitous, facultative pathogenic environmental bacteria found in soil and water, with transmission mainly occurring through inhalation or ingestion [1]. In immunocompetent individuals, NTM infections typically manifest as chronic pulmonary disease, skin and soft tissue infection, or cervical lymphadenitis—particularly in children. In contrast, immunocompromised patients, especially those with human immunodeficiency virus (HIV), have an increased risk for disseminated NTM infections [2].

To date, approximately 200 species and 13 subspecies of NTM have been identified [3]. Mycobacteria can be differentiated into rapidly growing and slow-growing mycobacteria (e.g., *Mycobacterium tuberculosis* and *Mycobacterium avium*). Rapidly growing mycobacteria are nontuberculous mycobacteria that grow in culture within 7 days, commonly including *M. abscessus*, *M. fortuitum*, and *M. chelonae*. They are found in the environment (soil and water) and can cause skin, soft tissue, lung, and device-related infections, especially after surgery or in immunocompromised patients. Treatment is difficult because they are often drug-resistant and require prolonged combination antibiotic therapy rather than standard TB drugs. Rapidly growing mycobacteria are not very common in children, but they can occur, especially after invasive procedures in children with underlying risk factors. Most pediatric cases involve skin and soft tissue infections after trauma, surgery, injections, or contaminated medical/cosmetic procedures. Pulmonary disease from rapidly growing mycobacteria is rare in healthy kids but may be seen in children with cystic fibrosis or chronic lung disease. Disseminated infection is uncommon and mainly affects immunocompromised children [4].

The most commonly implicated slow-growing pathogens in human disease are members of the *Mycobacterium avium* complex, *Mycobacterium kansasii*, *Mycobacterium xenopi*, and *Mycobacterium abscessus* [2].

In pediatric patients, NTM infection predominantly involves the cervicofacial lymph nodes. The clinical presentation is characteristic, beginning with a painless mass in the neck or parotid region, which over weeks to months evolves into an erythematous to violaceous lesion that may spontaneously fistulate, ulcerate and drain. Typically, the affected child is otherwise asymptomatic, with barely elevated inflammatory laboratory parameters (e.g., C-reactive protein (CRP), leukocytosis) [5]. Differential diagnosis of cervical lymphadenopathy in children is usually reactive or infectious lymphadenopathy, most often caused by viral infections (e.g., Epstein–Barr virus (EBV), cytomegalovirus (CMV) or bacterial pathogens like Staphylococcus and Streptococcus). Less commonly, autoimmune diseases (e.g., Kawasaki disease) or malignancies such as lymphoma should be considered, especially if there are systemic symptoms or firm, fixed nodes [6].

Diagnosing NTM disease remains particularly challenging. A major obstacle is the lack of consensus on diagnostic criteria and treatment strategies, which complicates clinical decision-making. Furthermore, the slow and often difficult cultivation of mycobacteria from clinical specimens frequently results in delayed diagnosis and therefore postponed initiation of appropriate antimicrobial therapy. In this context, sonographic evaluation of the affected regions represents an invaluable first-line diagnostic tool: it is rapidly accessible, non-invasive, and does not involve exposure to ionizing radiation, which is particularly advantageous in pediatric patients. A conservative treatment with antibiotics, as well as a watch-and-wait strategy, also has a high chance of cure and should be considered as a treatment option [5,7,8].

Taken together, there is no consensus on the treatment of NTM-associated lymphadenopathy (LAP). Surgical procedures, in comparison to conservative treatment with antibiotics or watchful waiting, appear to be advantageous in terms of disease duration and postoperative cosmetic outcome, but the indication must be carefully weighed against the risk of damage to the facial nerve [5,7,8,9].

## 2. Materials and Methods

We conducted a retrospective analysis of all patients diagnosed with NTM-associated cervical LAP who were treated at the Department of Otorhinolaryngology, Medical University of Innsbruck, between January 2010 and December 2024. The Department of Otorhinolaryngology comprises three wards, including a dedicated pediatric ENT unit, with a total capacity of 24 adult inpatient beds, 2 day-care beds, and 7 pediatric beds. In 2025, the department recorded 9253 inpatient admissions (midnight census) and 17,907 outpatient visits. Relevant cases were identified through the hospital’s electronic medical records system using a combination of ICD-10 codes related to NTM infections (ICD-Code A31.9, L04.0 and L04.1) and procedural codes, with a particular emphasis on presentations involving cervical LAP.

This study was approved by the review board of the Medical University of Innsbruck, Austria. All procedures conducted in this study involving human participants were in accordance with the ethical standards of the institutional review board and with the Helsinki Declaration of 1964 and its later amendments or comparable ethical standards.

Various patient and disease parameters were analyzed. These parameters included demographic data such as gender, age at diagnosis, BMI, epidemiological background, imaging findings (e.g., ultrasound, magnetic resonance imaging (MRI), diagnostic modality and duration), time to definitive diagnosis, identified NTM species, laboratory parameters (C-reactive protein [CRP] and erythrocyte sedimentation rate [ESR]), number of affected lymph nodes, treatment approach, and follow-up information.

These data were obtained from inpatient medical records and supplemented with information from paper-based charts. Relevant clinical variables were determined based on a systematic review of the literature using the PubMed database.

A systematic literature review was performed to define relevant variables and support the evaluation process. Clinical assessment included ultrasonography and MRI, fine-needle aspiration biopsy (FNA), microbiological testing using both liquid and solid culture media, and either surgical intervention or conservative antibiotic therapy. Microbiological analyses were carried out in specialized laboratories, and treatment decisions were made through interdisciplinary consensus.

In the absence of microbiological confirmation, the diagnosis of NTM-associated lymphadenitis was based on the characteristic clinical and radiological presentation. Typically, children present with a slowly progressive unilateral swelling in the submandibular or preauricular region, frequently accompanied by violaceous skin discoloration. Systemic symptoms such as fever, night sweats, or weight loss are usually absent, and patients are generally in good overall condition.

Ultrasonography commonly demonstrates necrotizing lymphadenitis with intranodal liquefaction, abscess formation, and occasionally cutaneous fistulization.

Immunological tests primarily developed for tuberculosis diagnostics have limited discriminatory value, as tuberculin skin testing and interferon-γ release assays may yield positive results in NTM infection. Therefore, these tests are not considered suitable for establishing a definitive diagnosis.

Data analysis was conducted using IBM SPSS Statistics for Windows, Version 24.0 (IBM Corp., Armonk, NY, USA). Descriptive statistics were used to summarize baseline characteristics. Categorical variables are reported as frequencies and percentages, while continuous variables are presented as median and interquartile range (IQR).

## 3. Results

### 3.1. Patient Characteristics

A total of 22 patients with cervicofacial NTM-associated LAP were identified and treated at our hospital (Table 1) between January 2010 and December 2024. The median age was 1 year (IQR 2), with a predominance of female patients (59%). The median BMI was 16.1 kg/m^2^ (IQR 1.7). The majority of patients resided in rural areas (91%), defined as smaller towns and market municipalities within the district of Innsbruck-Land, whereas only 9% were from urban settings, represented by the cities of Innsbruck and Bolzano. Further patient characteristics are summarized in Table 1. The median duration of symptoms prior to diagnosis was 7.9 weeks (IQR 7.6).

### 3.2. Diagnostic Methods

**Figure 1:**
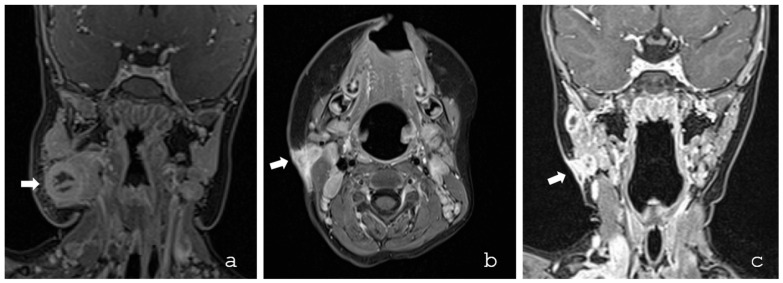
MRI in two patients with cervical NTM lymphadenopathy. (**a**) Coronal T2-weighted MRI image of a 1.5-year-old male patient demonstrated a necrotic lymph node in the right submandibular region (arrow). The lesion appears as a well-defined mass with central hyperintensity consistent with necrosis and a peripheral hypointense rim, suggestive of capsule formation or inflammatory reaction. The surrounding soft tissues exhibit mild edema, but no signs of infiltration into adjacent muscular structures are present. The imaging characteristics are indicative of necrotizing lymphadenitis. (**b**,**c**) MRI in a 2-year-old male patient with cervical NTM lymphadenopathy showed an ulcerating lymph node (arrows) measuring 15 mm immediately caudal to the right parotid gland or ventral to the sternocleidomastoid muscle with only tiny necrotic parts and with broad cutaneous infiltration ((**b**) axial, (**c**) coronal).

**Figure 2:**
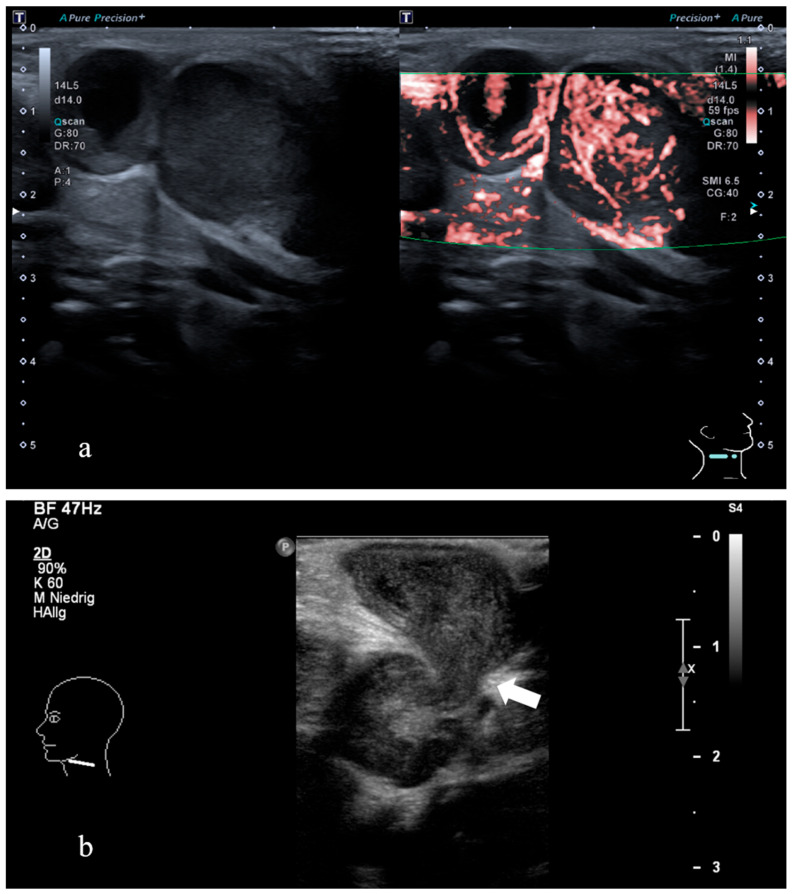
Ultrasound of a 2-year-old with cervical NTM lymphadenopathy. According to the clinical presentation, ultrasound showed a hypoechoic necrotic lymph node with irregular margins and irregular perfusion (**a**) and a fistulous tract consistent with skin perforation ((**b**), arrow) in level 2.

All patients were evaluated by a pediatrician and an otorhinolaryngologist. The diagnosis was suspected based on a characteristic clinical presentation. None of the patients had a known immunodeficiency or relevant predisposing risk factors. Cervical ultrasound was performed in all patients (22/22; 100%), while additional MRI was undertaken in 12/22 cases (55%) to provide detailed anatomical information and complement sonographic findings (Figure 1 and Figure 2).

The median number of affected lymph nodes per patient was 2 (IQR 1), with a median short-axis diameter of 13 mm (IQR 8) and a median long-axis diameter of 21 mm (IQR 12.75) (Table 2).

Lymphadenopathy was most frequently observed in the submandibular region (Level IB; 54.1%), followed by jugulodigastric nodes (Level IIA/B; 24.3%), preauricular/parotid nodes (13.5%), and submental nodes (Level IA; 8.1%). Microbiological confirmation was achieved in 14/22 patients (64%) using culture-based methods, with liquid culture medium yielding slightly higher detection rates than solid medium (13 vs. 10 positive results). Culture results were typically available within 7.9 weeks (IQR 7.6). The most frequently identified species was *Mycobacterium avium* (n = 12), followed by single isolates of *Mycobacterium kansasii* and *Mycobacterium malmoense*. Antimicrobial susceptibility testing, when available, showed high susceptibility to clarithromycin, whereas resistance to standard antituberculous agents such as rifampicin, isoniazid, and pyrazinamide was frequently observed (Table 3 and Table 4).

In 8/22 patients (36%), no pathogen could be identified. FNA was performed in 3/22 patients (14%), but yielded no mycobacterial growth. In a further 3/22 patients (14%), histological confirmation was not pursued; diagnosis in these cases was based solely on morphological imaging findings in conjunction with clinical presentation. The median time to diagnosis verification was 7.9 weeks (IQR 7.6). None of the patients presented with fever at diagnosis. Occasional febrile episodes were observed following surgical procedures or during unrelated respiratory infections and were therefore not considered disease-specific. Inflammatory laboratory parameters were only mildly elevated at presentation. The median C-reactive protein (CRP) level was 0.25 mg/dL (IQR 0.47), and the median erythrocyte sedimentation rate (ESR) was 15 mm/h (IQR 13.5). Following initiation of therapy, both CRP and ESR declined to median values of 0.06 mg/dL (IQR 0.09) and 9.5 mm/h (IQR 9), respectively. For this analysis, the first available CRP and ESR values were used, obtained either at our institution or from external hospitals; several patients had received outpatient antibiotics prior to referral (Table 5). 

In our cohort, immunological testing demonstrated heterogeneous findings. A positive tuberculin skin test and/or ELISPOT was observed in a minority of patients. In one case, simultaneous positivity of both assays was attributed to an immune response to *Mycobacterium kansasii*. In five additional patients, the tuberculin skin test was positive, while ELISPOT remained negative. Patients receiving rifampicin therapy underwent regular laboratory monitoring, including liver function tests, in line with standard clinical practice; these tests were performed locally by the patients’ general practitioners or pediatricians.

### 3.3. Therapy

**Figure 3:**
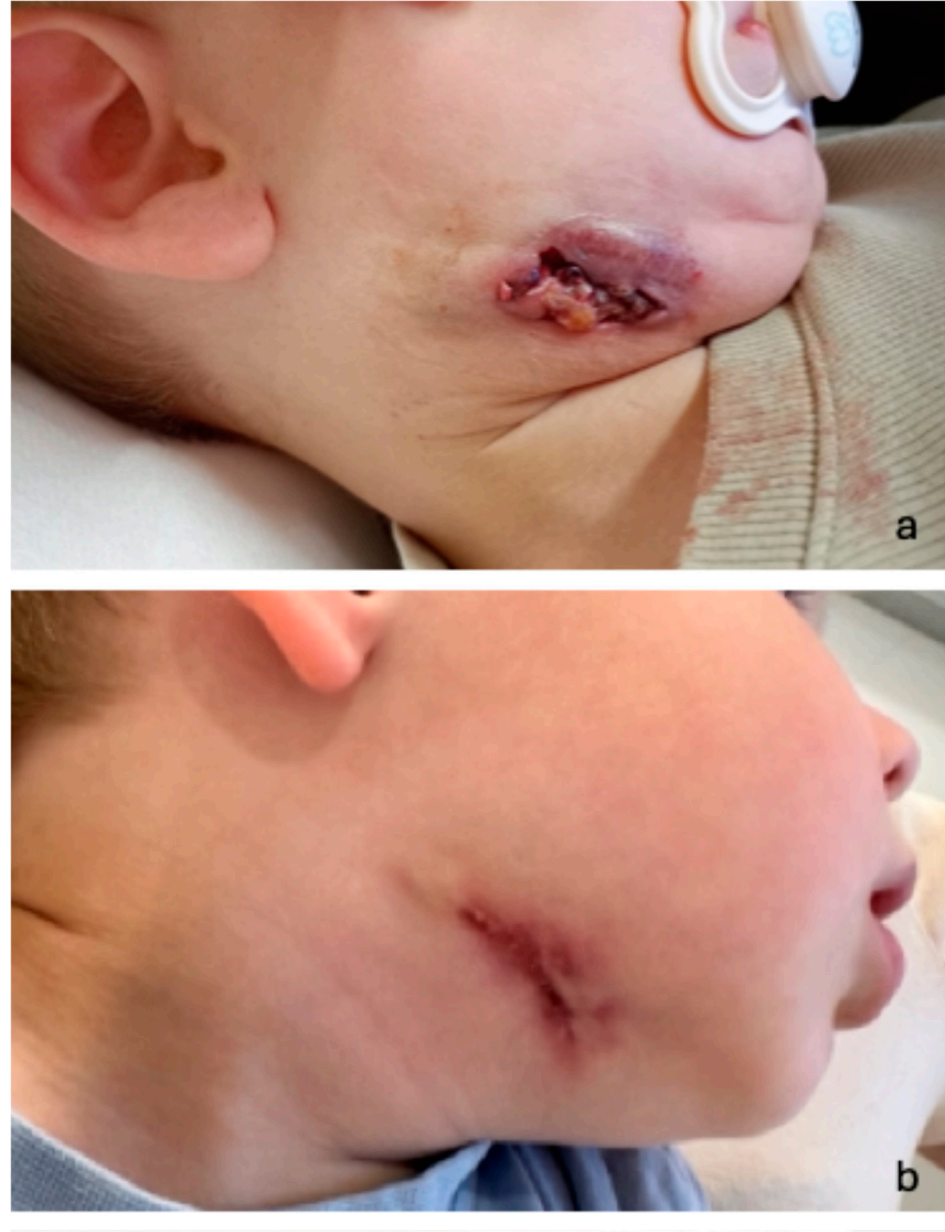
Clinical presentation of a 2-year-old boy with cervical NTM lymphadenopathy ulcerating lymphadenopathy with cutaneous fistulation at initial presentation (**a**) and at follow-up 4 months after surgery (**b**). Potential differential diagnoses comprise bacterial lymphadenitis, tuberculosis, or malignant conditions such as lymphoma.

Treatment consisted of a combination of clarithromycin and rifampicin, administered for a duration of 3–6 months. The length of antibiotic therapy was individualized according to clinical course, with discontinuation upon complete resolution of the disease. Surgical intervention (Table 2, treatment overview) was performed in 16/22 patients (73%), particularly in cases with pronounced clinical symptoms (Figure 3).

Median follow-up duration was 4 months (IQR 9.8); during follow-up, 17 patients demonstrated marked regression of clinical signs and imaging findings, typically within six months. One patient remains under follow-up at our institution, showing clear signs of improvement. Three additional patients are being monitored locally by their primary care physicians, and one patient was lost to follow-up.

## 4. Discussion

In our cohort, the majority of cases occurred in female infants with a median age of approximately one year, predominantly presenting with unilateral, painless cervical lymphadenopathy, often accompanied by violaceous skin discoloration and a tendency toward fistula formation. A notable predominance of patients from rural regions was also observed.

The predominance of patients from rural areas in our cohort aligns with the widespread environmental distribution of NTM in soil, dust, air, vegetation, and natural and treated water systems (often via biofilms), as well as in certain foods, raw milk, and wildlife [10,11]. Children in rural environments may therefore have greater exposure to these reservoirs, potentially increasing infection risk. However, this remains speculative and warrants confirmation through large-scale, population-based studies incorporating detailed environmental and demographic data. The higher incidence of NTM LAP in female patients remains poorly understood. While environmental exposure to soil-dwelling NTM has been proposed as a potential risk factor, Kuntz et al. suggest that the consistently higher frequency of NTM infections in females across diverse geographic and climatic regions more likely reflects immunological factors or host–pathogen interactions rather than environmental exposure alone [12].

In all patients, cervical ultrasonography was performed as the initial diagnostic modality, providing rapid, non-invasive, and radiation-free evaluation without the need for sedation—an important consideration in pediatric patients. Ultrasound findings were sufficiently characteristic to raise strong suspicion of NTM LAP, and MRI was employed in selected cases to delineate anatomical details preoperatively or to exclude differential diagnoses such as malignant disease, tuberculosis, or lymphoma.

FNA was performed in three patients but failed to yield microbiological confirmation in any case. Furthermore, FNA was associated with poorly healing fistulas and wound complications, consistent with reports of reduced cure rates following such procedures compared to conservative management [7]. These findings support current recommendations to avoid FNA and other invasive measures such as incision, drainage, or curettage. Definitive diagnosis relies on positive mycobacterial culture and/or polymerase chain reaction (PCR) from purulent or aspirated material. In our study, culture-based methods were used in the majority of cases, with a median time to pathogen identification of 7.9 weeks, most frequently from liquid cultures. As reported in the literature, mycobacterial cultures may require up to six weeks for pathogen identification and show a 67.2% sensitivity rate, with faster-growing rates in liquid cultures [13,14]. No pathogen was identified in 36% of patients, necessitating diagnosis based on clinical and radiomorphological findings alone in some cases. The relatively low detection rates and prolonged culture times reflect the inherent challenges of cultivating mycobacteria, attributable to their lipid-rich, impermeable outer membrane, slow growth, and biofilm formation [15,16,17].

Currently available rapid diagnostic tests, such as those commonly used for tuberculosis, have no diagnostic value for NTM infections. The MMT is frequently positive in both tuberculosis and NTM infections and may also yield positive results following Bacillus Calmette-Guérin (BCG) vaccination. In contrast, interferon-γ release assays (IGRAs; e.g., Quantiferon and enzyme-linked immunosorbent spot (ELISPOT)) are highly specific for *M. tuberculosis* and are generally negative in NTM infections, with the exception of *M. kansasii*, *M. marinum*, and *M. szulgai*, which express the antigens 6 kDa Early Secreted Antigenic Target (ESAT-6) and 10 kDa culture filtrate antigen CFP-10 (CFP-10). Thus, IGRAs offer higher specificity than the MMT, particularly in BCG-vaccinated or NTM-exposed populations [18,19,20].

In our cohort, immunological testing demonstrated heterogeneous results. A minority of patients showed positive findings in tuberculin skin testing (TST/MMT) and/or ELISPOT. In one case, simultaneous positivity of both assays was attributed to an immune response to *Mycobacterium kansasii*, whereas in five additional patients, the tuberculin skin test was positive while ELISPOT remained negative. These discordant results underscore the well-known diagnostic difficulties in pediatric NTM lymphadenitis.

Microbiological confirmation is frequently hampered by the slow growth and limited sensitivity of cultures, particularly in paucibacillary disease. Furthermore, immunological assays may yield reactive or inconclusive findings and therefore cannot reliably differentiate tuberculosis from NTM infection. Consequently, in routine clinical practice, diagnosis often relies on the combination of a characteristic clinical presentation, supportive imaging findings, and exclusion of alternative etiologies. Our observations reflect this real-world scenario and are consistent with previously published pediatric case series [7,21].

Surgical intervention was performed in most patients and proved to be effective [5]. Conservative strategies, including antibiotic-only regimens or watchful waiting, are also described in the literature. In our cohort, treatment decisions were based on individual risk profiles: patients with increased risk of facial nerve injury were treated primarily with combined antibiotic therapy (rifampicin and clarithromycin for 3–6 months), while surgical excision was preferred when diagnosis remained uncertain or when immunodeficiency or malignancy could not be excluded. All patients receiving surgery also underwent adjuvant antibiotic therapy. The observed susceptibility pattern supports the current preference for macrolide-based regimens in pediatric NTM lymphadenitis.

Source control represents an important principle in the management of NTM lymphadenitis. Several studies have suggested that complete surgical excision or drainage of the affected lymph node may, in selected cases, be sufficient to achieve a cure, particularly in infections caused by rapidly growing mycobacteria. However, treatment strategies remain heterogeneous, and many centers favor a combination of surgery and antimicrobial therapy, especially in advanced or complicated disease.

The duration of antibiotic therapy in our cohort was highly variable (median 45.5 days, IQR 143.3) and was discontinued upon complete resolution of symptoms. This variability mirrors findings from previous studies, which typically report treatment durations of around three months but emphasize the absence of standardized recommendations and the influence of patient-specific factors on therapy length.

Emerging biomarkers, such as serum neopterin and the urinary neopterin/creatinine ratio, may offer valuable tools for monitoring disease activity and treatment response in pediatric patients with nontuberculous mycobacterial (NTM) cervical lymphadenitis. Neopterin, a marker of cellular immune activation, has been studied in various infectious and inflammatory conditions and may reflect ongoing immune response even in the absence of overt clinical symptoms. Monitoring neopterin levels could potentially support the management of patients undergoing different therapeutic strategies, including surgical excision, antibiotic therapy alone, or a conservative watch-and-wait approach. Although our current study did not include measurements of serum or urinary neopterin, future prospective studies incorporating these biomarkers may provide additional insights into disease monitoring and individualized treatment planning in this patient population [22].

### 4.1. Limitations

This study is limited by its retrospective design and relatively small sample size, which may affect the generalizability of the findings. Microbiological confirmation was not achieved in over one-third of cases, necessitating reliance on clinical and imaging criteria for diagnosis. Additionally, variability in treatment duration and approach reflects the lack of standardized therapeutic guidelines for pediatric NTM-associated cervical lymphadenitis. For the analysis of inflammatory markers, the first available CRP and ESR values were used, regardless of whether they were obtained at our institution or during prior evaluations at referring centers. As some patients had already received antibiotic therapy before presentation, these parameters may not fully represent the untreated baseline inflammatory status.

Furthermore, precise quantification of the number of affected lymph nodes was sometimes difficult because nodes were frequently conglomerated. Finally, clinical symptoms were not documented in a standardized fashion in all records, limiting the assessment of variables such as pain or drainage.

### 4.2. Summary

Our findings underscore the central role of ultrasonography as a first-line diagnostic tool in pediatric NTM lymphadenitis, offering reliable, rapid, and low-risk assessment while reducing the need for anesthesia. The considerable variability in antibiotic treatment duration observed in our cohort, consistent with heterogeneity in the literature, highlights the ongoing lack of standardized therapeutic guidelines. Future research should aim to establish evidence-based diagnostic and treatment algorithms to optimize patient outcomes and minimize the burden of invasive interventions and prolonged therapies.

## 5. Conclusions

NTM infection should be considered in the differential diagnosis of pediatric cervical lymphadenopathy. Microbiological confirmation remains challenging, and invasive diagnostic procedures, such as puncture or drainage, should be carefully weighed. Ultrasonography represents the preferred first-line imaging modality. In selected cases, conservative strategies, including observation, may be appropriate.

## Figures and Tables

**Table 1:** Patient characteristics, diagnostic procedures and management (*n* = 22).

Variable	Category	*n* (%)
Sex	Female	13 (59.1)
	Male	9 (40.9)
Age	Median (IQR), years	1 (2)
Anthropometric data	Height, median (IQR), cm	86.5 (18.5)
	Weight, median (IQR), kg	12.0 (5.8)
	BMI, median (IQR), kg/m^2^	16.1 (1.7)
Fever at diagnosis	Yes	0 (0)
Treatment	Conservative	6 (27.3)
	Surgery	16 (72.7)
Imaging	Neck ultrasound	22 (100)
	MRI	12 (54.6)
Diagnostic classification	Culture confirmed	14 (63.6)
	Clinically diagnosed	8 (36.4)
Immunological tests	Positive TST/MMT	6 (27.3)
	Positive ELISPOT	1 (4.5)
	TST positive/ELISPOT negative	5 (22.7)

**Table 2:** Sonographic characteristics of cervical lymph nodes (*n* = 22).

Variable	Category	Value/*n* (%)
Number of affected lymph nodes	Median (IQR)	2 (1)
Lymph node diameter	Short axis, median (IQR), mm	13 (8)
	Long axis, median (IQR), mm	21 (12.8)
Localization (sonographic level)	Preauricular/parotid	5 (13.5)
	Submental (Level IA)	3 (8.1)
	Submandibular (Level IB)	20 (54.1)
	Jugulodigastric (Level IIA/B)	9 (24.3)

**Table 3:** Overview of treatment modalities.

Variable	Details	*n* (%)
Antibiotic therapy only	Clarithromycin + Rifampicin, 3–6 months	6 (27.3)
Surgery only	Complete excision, no antibiotics	-
Surgery + antibiotics	Excision + Clarithromycin + Rifampicin	16 (72.7)
Duration of antibiotic therapy	Median 45.5 days (IQR 143.3)	-
Treatment outcome	Regression within 6 months	17 (77.3)

**Table 4:** Antimicrobial susceptibility patterns of available NTM isolates. Percentages are calculated based on the number of isolates tested for each antibiotic.

Antibiotic	Tested *n*	Susceptible *n* (%)	Resistant *n* (%)
Clarithromycin	7	7 (100.0)	-
Rifampicin	6	-	4 (66.7)
Rifabutin	7	1 (14.3)	4 (57.1)
Ethambutol	7	1 (14.3)	4 (57.1)
Amikacin	3	2 (66.7)	1 (33.3)
Streptomycin	6	-	5 (83.3)
Moxifloxacin	7	1 (14.3)	2 (28.6)
Isoniazid	6	-	4 (66.7)
Pyrazinamide	4	-	4 (100.0)
Linezolid	1	-	1 (100.0)
TMP/SMX	2	-	-

**Table 5:** Inflammatory parameters at presentation and after initiation of therapy.

Variable	Time Point	Median (IQR)
CRP, mg/dL	At initial presentation	0.25 (0.47)
	After initiation of therapy	0.06 (0.09)
ESR, mm/h	At initial presentation	15 (13.5)
	After initiation of therapy	9.5 (9)

## Data Availability

The data presented in this study are not publicly available due to ethical and data protection restrictions. Data may be made available from the corresponding author upon reasonable request and with permission of the Institutional Review Board.
